# Recurrent Respiratory Papillomatosis (RRP)—Juvenile Onset

**DOI:** 10.4137/cmo.s698

**Published:** 2008-07-18

**Authors:** Sara W. Dyrstad, Krishna A. Rao

**Affiliations:** Department of Internal Medicine, Southern Illinois University School of Medicine, 415 North Ninth Street, Post Office Box 19678, Springfield, IL 62794-9678 U.S.A

**Keywords:** human papilloma virus, respiratory papillomatosis, cancer

## Abstract

In this article, we describe the treatment of long standing juvenile-onset recurrent respiratory papillomatosis (JORRP) with eventual transformation to carcinoma in a patient who lived to the age of 73. Treatment modalities consisted of bronchoscopy and local excision initially. Later, YAG and CO2 laser debulking were used. Radiotherapy, chemotherapy with carboplatin (300 mg/m2) and 5-FU (600 mg/m2), oral methotrexate (5–7.5 mg/week), pegylated Interferon, indole-3-carbamide, and intralesional cidofovir were also utilized in the treatment of this patient. Except for methotrexate, each of the treatment regimens used in this patient, initially decreased growth of the papillomas and improved symptoms experienced by the patient. Interestingly, we found that this patient’s long standing JORRP initially responded to a chemotherapy regimen of 4 cycles of carboplatin (300 mg/m2) and 5-FU (600 mg/m2) as well. Ultimately, the disease became resistant to all forms of treatment and progressed. The patient eventually succumbed to the disease after an approximate 77 year course.

## Background

Recurrent Respiratory Papillomatosis (RRP) is caused by certain strains of the human papilloma virus, HPV-6 and HPV-11.(Auborn, 2002) This highly morbid disease can begin in childhood or adulthood. However, only 4 to 6 per 100,000 people exposed to HPV-6 or HPV-11 develop this disease. In children, this disease normally presents before the age of 5 and is referred to as juvenile onset RRP (JORRP). JORRP is usually thought to be vertically transmitted to the child during childbirth, although placental transmission has also been reported (Wang, 1998).

In adults, this disease commonly presents in the fourth decade of life and is referred to as adult onset RRP (AORRP). The cause of AORRP is unknown. But, sexual transmission is suspected. Males have a higher risk (4-fold) when compared to females in developing AORRP.(Auborn, 2002) RRP usually develops at the junction between squamous and columnar epithelial cells, such as the vocal cords, subglottis, laryngeal side of epiglottis, and trachea. Altered immune response to HPV proteins has been hypothesized as a factor involved in the development of RRP.(DeVoti et al. 2004) Because pregnancy has been found to enhance disease progression, hormonal factors are thought to be involved in the pathogenesis of RRP. The morbidities associated with any form of RRP are due to bronchial obstruction and the use of procedures and multiple drug regimens necessary to deter the growth of the papillomas.(Auborn, 2002)

Treatment for RRP normally consists of initial debulking procedures using laser, microsurgical, excision, or microdebrider to improve the airway and voice. Some children can require frequent removal, with intervals between surgery sometimes 3–4 weeks and occasionally even shorter.(Auborn, 2002) Interferon therapy and antivirals have been shown only to slow the progression of the papilloma growth, without demonstrating a cure. It has been suggested that tracheostomy placement may cause further spread of the papillomas into the trachea and bronchi; however it is equally likely that tracheotostomy placement is simply a marker of a more aggressive form of the disease. A small subset of patients with either JORRP or AORRP develop malignant degeneration to squamous cell carcinoma. The use of irradiation and other DNA damaging agents have been implicated as risk factors for malignant transformation as well.(Auborn, 2002)

Chemotherapeutics currently being studied as treatment options for RRP include indole-3-carbinol, photodynamic therapy (PPT), interferon, retinoid therapy, and HPV vaccination, used alone or in combination with surgical excision. None has been shown to cure the disease, but chemotherapy can prolong the duration of symptom free intervals. For each drug, the decision to treat must be weighed against the potential risk of malignant degeneration.

## Case Report

W.E. a 75-year-old female with a long history of RRP in the larynx and trachea that required multiple excisions of the papillomas. The patient claimed to have had papillomas present in her larynx ever since the age of 2. Her first documented surgery for RRP occurred at age 22. By 2000 (age 72), she had undergone multiple surgeries for excision of the papillomas, estimated to be approximately 60–70. She had no known history of tobacco or alcohol use. In 2000, the papillomas became rapidly progressive with obstruction, and histopathologic examination revealed squamous cell carcinoma *in situ. She* underwent external beam radiation totaling 6200 rads to the region encompassing the larynx, trachea and the upper mainstem bronchus. Initially, the tumors responded to the therapy, but regrew rapidly in 2001, requiring further excision. She was treated with 4 cycles of carboplatin (300 mg/m^2^) and 5-FU (600 mg/m^2^) given every 3 weeks. Initially, W.E. responded to therapy, but the disease progressed again, requiring laser excision. Next, she was treated with methotrexate initially at 7.5 mg orally each week, decreasing to 5 mg orally weekly due to mucosal dryness, but, the papillomas continued to grow rapidly and a tracheotomy was required. A chest x-ray and CT scan revealed pulmonary nodules suspicious for metastases. The patient was lost to follow-up. In August of 2003, W.E. returned with symptoms of airway obstruction after a long hiatus with no therapy and received 2 cycles of continuous infusion 5-FU for five days at a dose of 500 mg/m^2^ per day. A repeat CT of chest and neck revealed mild interval progression of a few pulmonary nodules with no interval change in most of the lesions. She continued to be treated with frequent surgery and one month after treatment with 5FU, biopsy in September, 2003, revealed invasive squamous cell carcinoma of the larynx. A CT-guided biopsy of her enlarging left lung base nodule revealed a high-grade squamous cell dysplasia. A laryngectomy was performed in October 2003 and chemotherapy was resumed with carboplatin and 5-FU. The chemotherapy was discontinued towards the end of February 2004 after the patient experienced dizziness, tiredness, nausea, anorexia and eye pain. In February 2004, pegylated Interferon was started at a dose of 50 mcg per week, which she tolerated very poorly. She was given Indole-3 carbamide (400 mg orally daily) and in March 2004, she had debridement of peristomal papillomas with injection of cidofovir, as well as bronchoscopy with Argon laser coagulation of papillomas in her distal airway. In July 2004 for stomal obstruction secondary to recurrent/worsening laryngeal tracheobronchial papillomatosis, an Argon laser coagulation (60minutes) was performed and the debulking of the tumor allowed mild relief of her symptoms. She developed a left neck mass, and a fine needle aspiration of the mass showed keratinizing squamous cell carcinoma. She was not a candidate for further chemotherapy or radiation and was referred to hospice and expired in October 2004. Over the duration of her illness, medical input was obtained from Hematology-Oncology, ENT, Radiation Oncology and Pulmonary services. Her case was presented and further discussed at both ENT and pulmonary tumor boards and resulted in treatment plans derived from the consensus opinion.

## Discussion

Surgery, radiation therapy, chemotherapy, Indole-3-carbamide, Interferon (INF), and cidofovir were used in this case of JORRP, each of which was only transiently effective. Endoscopic removal remains the mainstay of treatment for respiratory papillomatosis. Surgical techniques include laser, cold steel, and microdebrider. Repeated surgery can lead to scarring and stenosis. Radiation therapy was used as a treatment of desperation for this patient to prevent impending obstruction after detection of squamous cell carcinoma *in situ, with an understanding that the radiation might promote future transformation to invasive malignancy*. Radiation therapy has been implicated as causing malignant transformation and should usually be avoided unless malignancy has been diagnosed. In this particular case, radiation therapy was beneficial in short term management of obstructive symptoms, although it may have accelerated progression to malignancy in the long term.(Auborn, 2002)

Indole-3-carbinol/diindolylmethan (I3C/DIM), commonly found in cruciferous vegetable, is the most benign adjuvant therapy for RRP.(Rosen and Bryson, 2004) The compounds I3C or DIM, a dimer of I3C, induces 2-hydroxylation of estradiol, forming 2-hydroxyoestrone, which is not estrogenic. HPV-6 and HPV-11 usually cause disease at transformation zones such as the cervix or vocal cords, which are areas that tend to be hormonally sensitive. I3C/DIM also has antiproliferative effects in papillomas and acts as a free radical scavenger.(Auborn, 2002) Rosen and Bryson (Rosen and Bryson, 2004) found I3C to be a successful treatment option for RRP, with its efficacy in children meriting further study. After complete surgical removal of papillomas, 11 (33%) patients had remission of papilloma growth requiring no further debridement procedures while on I3C. Ten (30%) patients had a decreased papilloma growth. Twelve (36%) patients had no clinical response and no patients’ growth of papillomas increased. Rosen et al. concluded that I3C was safe and well tolerated.(Rosen et al. 1998) There are very few side affects with I3C/DIM treatment. For unknown reasons, there are subsets of people that do not respond to I3C, and this could be related to differences in estrogen metabolism. (Rosen and Bryson, 2004)

Interferon-α therapy, is used as an adjuvant therapy and has been shown to slow the recurrence rate of RRP. The effectiveness of interferon appears to be dose related. INF-α induces cells to become resistant to various viruses, and decreases growth of certain cells. INF-α can cause flu-like symptoms and problems with liver function tests. In 1988, Kashkima found that interferon alpha-n1 (Wellferon) was an effective adjuvant to surgery in treating RRP. In a 12-month randomized crossover study, 66 patients with clinically severe JORRP were evaluated. Statistically, significant improvement occurred in patients receiving INF-α n-1.(Kashima et al. 1988) Healy demonstrated in 1988 that interferon is neither curative nor of substantial value when used as an adjuvant agent in long-term management of RRP. In this study, 123 patients were randomly assigned to receive surgery plus INF, or surgery alone. Healy found that the initial effects of interferon were not maintained.(Healy et al. 1988) It has been shown that disease severity increases after cessation of INF-α. Because of the higher incidence of side affects, INF-α tends to be used either after a patient has failed I3C therapy or along with I3C.(Auborn, 2002) In this case study, INF-α and I3C were used simultaneously.

Cidofovir is a nucleoside analogue that disrupts viral DNA synthesis and angiogenesis and promotes apoptosis, and is particularly useful in RRP as it is a HPV-driven process. Cidofovir is normally used intralesionally for adjuvant treatment of RRP.(Snoeck, 1998) This decreases the chance of kidney damage that can occur with systemic cidofovir.(Auborn, 2002) In 1999, Pransky found that 4 out of 5 pediatric patients with severe RRP responded to intralesional cidofovir treatment. One patient was disease-free and 3 patients had a dramatic response at their 9-month follow-up visit.^7^ The use of Cidofovir was started late in the course of this patient’s disease process, and may perhaps have proved more beneficial if started earlier in her disease course.

Understanding the molecular biology of RRP has advanced the adjuvant treatment options with concurrent surgical/laser debridement. In 1999, Bonagura found that patients with RRP had an elevated percentage of CD8 (+), CD28 (−) T-cells. They also found that many of the patients tested expressed an increase in the TH2-like cytokine mRNA in response to autologous papilloma tissue. Bonagura’s data suggested that patients with RRP do not form an adequate T-cell response to HPV. These alterations in the immune system could lead to different levels of disease severity.(Bonagura et al. 1999) In 1998, Rady discovered that only integrated forms of HPV-11 sequences were found in malignant tissue samples, and through molecular genetics demonstrated the allelic loss of INF-β gene and a mutation of p53 tumor suppressor gene in only malignant lesions. Rady also found that the p53 genetic mutation correlated with the integration of HPV-11 in malignant tissues.(Rady et al. 1998) In 2004, DeVoti explored the cytokines expressed by peripheral blood mononuclear cells and T-cells from RRP patients that were exposed to purified HPV early proteins E6 and E7. DeVoti’s results suggested that HPV-11 E6 alters the expression of IL-10 and INF-γ, towards increased expression of IL-10 versus INF-γ. They also found a hyporesponsiveness of TH1-like cytokines in response to E6 in patients with more severe disease. DeVoti suggested that it was this alteration in the immune response that contributed to the inability of a patient’s immune system to control RRP.(DeVoti, Steinberg, Rosenthal, Hatam, Vambutas, Abramson, Shikowitz, and Bonagura, 2004)

In 1998, McKaig et al. noted the overall prevalence of HPV in head and neck tumors was 34.5% (416 of 1205 tumors) with the frequency of HPV in benign and precancerous lesions ranging from 18.5% to 35.9%. HPV positive tumors were also more often found to be the high risk types 16 and 18 (40% and 11.9% respectively). HPV positive tumors were detected in the oral cavity more often (59%), then the pharynx (43%) or larynx (33%). McKaig et al. concluded that the high prevalence of HPV suggested a potential etiologic role in head and neck cancer.(McKaig RG et al. 1998) Li et al. in 2004 looked at cell marker expression and presence of HPV in fifty cases of SCC of the tonsil. HPV, mostly type 16, was identified in 42% of cases with a statistical association between reduced expression of pRb and cyclinD1, overexpression of p16, and a younger patient age. Li et al. concluded that HPV-positive SCC of the tonsil has a distinct molecular pathway with from HPV-negative tumors.(Li W. et al. 2004) When looking at younger versus older patients with SCC of the head and neck, Sisk found the incidence of HPV to not be significantly different between the groups studied. Interestingly, Sisk et al. noted some survival advantage in patients bearing HPV-positive tumors relative to patients with HPV-negative tumors.(Sisk et al. 2000)

In this case report, it became evident that JORRP, with progression to squamous cell carcinoma, initially responded to multiple agents including chemotherapy regimens of carboplatin and 5-FU. It also became evident that JORRP then developed resistance to these agents, and progression of disease occurred. Photodynamic therapy (PDT), additional antiviral medications, HPV vaccination, retinoid treatment, and velcade are all novel agents developed to further control the progression of disease in RRP.

PDT uses a photosensitizing dye that the neoplastic cells selectively take up. These tissue regions are then exposed to a certain wavelength via an argon laser. This creates a singlet oxygen reaction resulting in an anti-angiogenic effect.(Auborn, 2002) In 1998, Shikowitz using Dihematoporphyrinether (DHE) PDT demonstrated in a randomized prospective trial that patients receiving either 3.25 mg/kg or 4.25 mg/kg dose showed notable improvement over the first year. Patients receiving the dose 4.25 mg/kg demonstrated a significantly larger decrease in papilloma growth rate. The improvement in a subset of people was maintained at the 3-year follow-up.(Shikowitz et al. 1998) DHE can leave patients sensitive to light for approximately 2 months. In 2005, Schikowitz used a newer PDT agent, meso-tetra hydroxyphenyl chlorine (mTHC), or Foscan, and it reduced the severity of laryngeal papillomas, but failed to maintain remission. Five of the 15 patients were in remission 12–15 months after treatment, but had disease reoccurrence after 3–5 years. Out of the 23 patients (ages 4–60 years) that initially began this study, only 15 people were available for follow-up. Shikowitz concluded that mTHC was not an optimal treatment.(Shikowitz et al. 2005)

Other antivirals such as acyclovir and ribavirin offer promise as additional adjuvant therapies for RRP resistant to other modalities. Acyclovir, a purine analogue, is preferentially activated by thymidine kinases encoded by herpes viruses and is then incorporated into viral DNA causing strand breakage. Although the papillomavirus does not preferentially activate thymidine kinase, there have been clinical responses to acyclovir in patients with RRP. The current hypothesis is that a co-infection with herpes virus may exist. Ribavirin, a guanosine analogue, inhibits GTP synthesis and prevents RNA synthesis. There is not much literature about the efficacy of using ribavirin in patients with RRP. However, a small randomized study in the United States demonstrated an increase in the intervals between surgeries.(Auborn, 2002)

The use of various vaccinations is aimed at both prevention and treatment of RRP. Therapeutic vaccinations can target a non-structural viral protein such as E6 or E7 for specific targeting HPV induced papillomas or enhance a non-specific immune system response locally by the presentation of a foreign antigen (e.g. mumps vaccine). There are some therapeutic and preventive vaccines that are being studied, such as TA-GW, which has a fusion protein of E7 and L2.(Auborn, 2002) In 2005, Pashley(Pashley, 2002) discovered that the mumps vaccination, in combination with serial laser debridement, was able to induce remission in children with RRP. After laser excision of the papilloma, the mumps vaccine was locally administered and in the pilot study Pashley found that remission occurred in 9 of 11 children (82%) with RRP after 1–10 vaccine injections, with a follow up of 5–10 years. In the other series, Pashley found that remission was induced in 29 of 38 patients (76%) by 4–26 injections, with a follow-up of 2–5 years. This subsequent series was composed of 18 children and 20 adults.(Pashley, 2002) Preventive vaccinations used against specific strains of HPV use recombinant viral L1 capsid proteins. The FDA has approved 1 HPV vaccine, Gardasil, which protects against infection from HPV 6, 11, 16 and 18. This vaccine should prevent infection with the 2 HPV subtypes (6, 11) most commonly associated with RRP, and theoretically enable primary prevention of the disease.(Harper et al. 2006)

Retinoids, both cis-retinoic acid or isotretinoin (accutane), have been used as adjuvant therapy in a small number of patients with RRP. Retinoids may inhibit growth of papillomas by inhibiting cell proliferation and angiogenesis. Results using cis-retinoic acid for RRP have demonstrated some success in decreasing disease progression. Toxicity of retinoids suspended 1 study which concluded the therapy to be ineffective.(Auborn, 2002)

Bortezomib (formerly PS-341), or Velcade, was approved by the FDA for treatment of multiple myeloma. Velcade inhibits the proteolytic activity of the proteosome complex in mammalian cells.(Kane et al. 2003) With Rady demonstrating a mutation of p53 suppressor gene found only malignant lesions and that a p53 genetic mutation correlated with the integration of HPV-11 in malignant tissues, Velcade may be a possible agent to use as adjuvant therapy in RRP.(Rady, Schnadig, Weiss, Hughes, and Tyring, 1998) HPV E6 binds to p53 and targets it for ubiquitin conjugation followed by proteasome degradation. Because of its potential to block p53 degradation intracellularly, Velacade is the first anti-cancer therapy to target the proteasome for inhibition and may be a valuable tool to treat later stage adult RRP. When tested in patients with multiple myeloma, the common adverse affects were asthenic conditions, nausea, vomiting, diarrhea, thrombocytopenia, and a peripheral neuropathy that was commonly painful.(Kane, Bross, Farrell, and Pazdur, 2003)

In this case study, we found that longstanding JORRP, ultimately progressing to invasive squamous cell carcinoma, initially responded to a chemotherapy regimen of 4 cycles of carboplatin (300 mg/m^2^) and 5-FU (600 mg/m^2^). We also found that the use of methotrexate did not halt the progression of the papillomas. Additionally, the use of carboplatin and 5-FU initially decreased the growth of the papillomas after squamous cell carcinoma *in situ* developed. However, the papillomas ultimately became resistant to therapy and the disease progressed to invasive carcinoma. Nonetheless, this case report offers the option of using a chemotherapy regimen for longstanding JORRP with progression to squamous cell carcinoma. With advancements in immune treatments, antivirals, and other novel agents such as PDT, further treatment options for longstanding JORRP do exist and HPV vaccination may make primary prevention of this disease possible.
